# Resilience as a Mediator Between Burnout and Health Among Nurses During the COVID‐19 Pandemic: A Cross‐Sectional Survey in Late 2021

**DOI:** 10.1002/nop2.70526

**Published:** 2026-04-14

**Authors:** Segundo Jiménez‐García, Esther Lázaro, Flores Vizcaya‐Moreno

**Affiliations:** ^1^ Clinical Nursing Research Group, Department of Nursing, Faculty of Health Sciences University of Alicante Alicante Spain; ^2^ Infectious Diseases Unit Elda University Hospital Alicante Spain; ^3^ Health Sciences Faculty Valencian International University Valencia Spain; ^4^ UNIE Universidad Madrid Spain

**Keywords:** burnout, COVID‐19, healthcare, mediation, mental health, nurses, occupational stress, physical health, quality of care, resilience

## Abstract

**Objective:**

To evaluate the mediating role of resilience in the relationship between burnout dimensions and the physical and mental health of nurses in December 2021 during the COVID‐19 pandemic.

**Design:**

Observational correlational study with a quantitative, cross‐sectional approach.

**Methods:**

A total of 256 nurses participated by completing an online survey in December 2021. Sociodemographic data were collected, and the Maslach Burnout Inventory–Human Services Survey, the Connor‐Davidson Resilience Scale, and the Short‐Form 12‐Item Health Survey version 2 were administered.

**Results:**

Resilience showed statistically significant indirect effects linking emotional exhaustion and depersonalisation to poorer mental health (negative indirect effects) and personal accomplishment to better mental health (positive indirect effect). Indirect effects involving physical health were smaller and less consistent: no indirect effect was observed for emotional exhaustion, whereas depersonalisation showed a small negative indirect effect and personal accomplishment showed a positive indirect effect through resilience. Resilience differed by sex and age, with men showing greater resilience.

**Conclusion:**

Findings are consistent with resilience being associated with mental health and statistically accounting for part of the association between burnout dimensions and mental health. Given the cross‐sectional design, causal direction cannot be inferred; resilience‐focused interventions should be evaluated in longitudinal and experimental studies.

**Implications for the Profession and Patient Care:**

Resilience is associated with better mental health and may represent a promising target for future intervention studies. Healthcare institutions could consider resilience‐support strategies alongside organisational measures to address burnout while evaluating their effectiveness using robust designs.

**Impact:**

- **What problem did the study address?** It explored how resilience can mediate the relationship between burnout and the physical and mental health of nurses during the COVID‐19 pandemic.
- **What were the main findings?** Resilience statistically accounted for part of the association between burnout dimensions and mental health, while indirect associations involving physical health were more heterogeneous and dimension‐specific.
- **Where and on whom will the research impact?** The findings will impact nurses in the Valencian Community and can extend to nursing professionals in similar contexts, informing practices and policies to improve well‐being and patient care.

**Reporting Method:**

The EQUATOR STROBE guidelines for cross‐sectional observational studies were followed, ensuring the quality and transparency of the report.

**Patient or Public Contribution:**

No patient or public contributed to the design or conduct of the study, the analysis or interpretation of the data, or the preparation of the manuscript.

## 1 Introduction

The COVID‐19 pandemic has had an unprecedented impact on health systems worldwide, especially affecting nursing staff (World Health Organisation 2020; The Lancet 2020). Nurses have been on the front lines of care, facing an intensified workload, lack of personal protective equipment (PPE), abrupt organisational changes, and challenges in their personal and family lives (Jiménez‐García et al. 2022; Shaukat et al. 2020). These factors have contributed to the increase of burnout syndrome in this group (García‐Campayo et al. 2016; Hu et al. 2020).

This study investigates whether resilience is associated with lower burnout‐related distress among nurses. Burnout is known to relate to adverse physical and mental health and can compromise the quality and safety of patient care; identifying potentially modifiable resources such as resilience may help inform future occupational health interventions.

The objective is to provide empirical data on the mediating role of resilience in the relationship between burnout and nurses' health in December 2021, a later stage of the pandemic. The results can guide occupational health policies to support staff exposed to sustained work‐related stress.

This work focuses specifically on late 2021, a period marked by persistent stress, accumulated fatigue, and structural strain within the healthcare workforce. These conditions provide context for interpreting associations between burnout, resilience, and health. Most previous studies have used cross‐sectional designs that only cover the early stages of the pandemic and do not capture its prolonged impact.

For instance, during the first wave, Luceño‐Moreno et al. (2020) reported high burnout levels among Spanish healthcare professionals; however, they did not examine resilience as an indirect pathway. Serrão et al. (2021) studied indirect associations through resilience among Portuguese workers but did not include later phases. García‐Izquierdo et al. (2018) assessed resilience as a moderating factor in a single care setting and did not examine physical health. By contrast, the present study applies regression‐based mediation models with bootstrap confidence intervals to examine indirect associations through resilience, incorporating both mental and physical health measures (SF‐12v2) and nurses working in diverse settings (hospital care, primary care, social healthcare, emergency services, and public health). This broader approach helps fill existing gaps and informs hypotheses for future intervention and longitudinal research on resilience and burnout in nursing staff.

### 1.1 Background

Burnout, described by Maslach and Jackson (1981), is characterised by three dimensions: emotional exhaustion, depersonalisation, and low personal accomplishment. During the pandemic, numerous studies have reported elevated levels of burnout among nursing professionals, associated with negative consequences for their physical and mental health, as well as for the quality of patient care (Morgantini et al. 2020; Pappa et al. 2020).

A previous study evidenced that 60.9% of nurses perceived a decrease in their personal well‐being during the first wave of the pandemic, attributable to changes in the work environment, inadequate use of PPE, and alterations in their personal lives (Jiménez‐García et al. 2022). Additionally, it was observed that nurses experienced high levels of stress and anxiety due to uncertainty and exposure to the virus (Kang et al. 2020; Luceño‐Moreno et al. 2020).

In this context, resilience has been described as a personal resource associated with better psychological outcomes under chronic work stress. Resilience is defined as the ability to adapt positively in the face of adversity, maintaining or recovering well‐being (Connor and Davidson 2003). Studies in nurses suggest that higher resilience is associated with lower burnout‐related distress and better mental health (García‐Izquierdo et al. 2018; Hart et al. 2014). Resilience has also been examined as an intervening variable linking work stressors with mental health and physical health outcomes (Mealer et al. 2012).

However, there is a gap in understanding how resilience can mediate the relationship between burnout and health in nurses during the advanced stages of the COVID‐19 pandemic. Although several studies have been conducted on the association between resilience and burnout, few have specifically included nurses (Connors et al. 2019; Guo et al. 2018; McCain et al. 2018; West et al. 2020; Yörük and Güler 2021).

Subsequent research has provided further evidence for indirect associations involving resilience in various clinical settings. Li et al. (2023) found that resilience mediated the association between social support and compassion fatigue among nursing and midwifery students during the COVID‐19 pandemic. In Canada, a study with 236 intensive care unit nurses during the second wave reported an indirect association of resilience with turnover intention linked to burnout (Rhéaume and Breau 2022). In Spain, Galdames et al. (2024) reported that resilience statistically accounted for part of the association between burnout and mental health among frontline nurses. These results point to the importance of examining the later stages of the pandemic and considering multiple levels of care. To our knowledge, no quantitative study has yet analysed both physical and mental health simultaneously or included nurses from diverse care settings in late 2021. This gap in evidence forms the basis for the present mediation analysis.

### 1.2 Theoretical Framework

The Transactional Model of Stress and Coping, first proposed by Lazarus and Folkman (1984) and later updated by Folkman (2013), describes how the stress response depends on both the characteristics of the external stimulus and the individual's sequential appraisal processes. In the primary appraisal, nursing professionals evaluate whether burnout, conceptualised as a psychological syndrome that develops as a prolonged response to chronic interpersonal stressors on the job (Maslach and Leiter 2016, 103), represents a threat, harm, or challenge to their well‐being. In the secondary appraisal, they consider the availability and effectiveness of personal resources and coping strategies. At this stage, resilience, defined as a set of cognitive, emotional, and social abilities that support self‐regulation, planned problem‐solving, and the use of support networks (Connor and Davidson 2003, 76), may function as a coping resource that could statistically account for part of the association between burnout dimensions and both mental and physical health. Accordingly, the present study tests an indirect association model in which burnout dimensions are linked to resilience, which in turn is linked to physical and mental health in a cross‐sectional context, without implying causal mechanisms.

## 2 Aim

The present study aims to evaluate the mediating role of resilience in the relationship between the dimensions of burnout and the physical and mental health of nurses in December 2021, during the COVID‐19 pandemic.

## 3 Materials and Methods

### 3.1 Study Design

This study adopted an observational correlational design with a quantitative and cross‐sectional approach. Data collection was conducted through a self‐administered online survey.

### 3.2 Participants, Sampling, and Eligibility Criteria

This study was conducted with nurses working at various levels of care in the public and private healthcare networks of the Valencian Community (Spain), recruited through convenience sampling in December 2021, during the COVID‐19 pandemic. Because the invitation was disseminated via multiple channels and assuming the most unfavourable proportion (*p* = 0.5) with a 95% confidence level, the final sample of 256 participants corresponds to an approximate precision of ±6% which was deemed sufficient for this exploratory study.

Eligibility criteria required nurses employed in public or private healthcare institutions in the Valencian Community during the data collection period, with access to the questionnaire, who provided written informed consent and did not report any health condition that might interfere with their completion.

### 3.3 Data Collection Procedure

An online survey was distributed over a 10‐day period in December 2021. This limited period sought to minimise possible biases derived from changes in perceptions and situations in a dynamic context. The survey was sent by email with a link to a Google Forms questionnaire, including a reminder 1 week later. Additionally, the link was disseminated through a WhatsApp distribution list and on social networks such as Twitter and Facebook. Informed consent was obtained from the participants, guaranteeing the confidentiality of their responses.

### 3.4 Measurement Instruments

**FIGURE 1:**
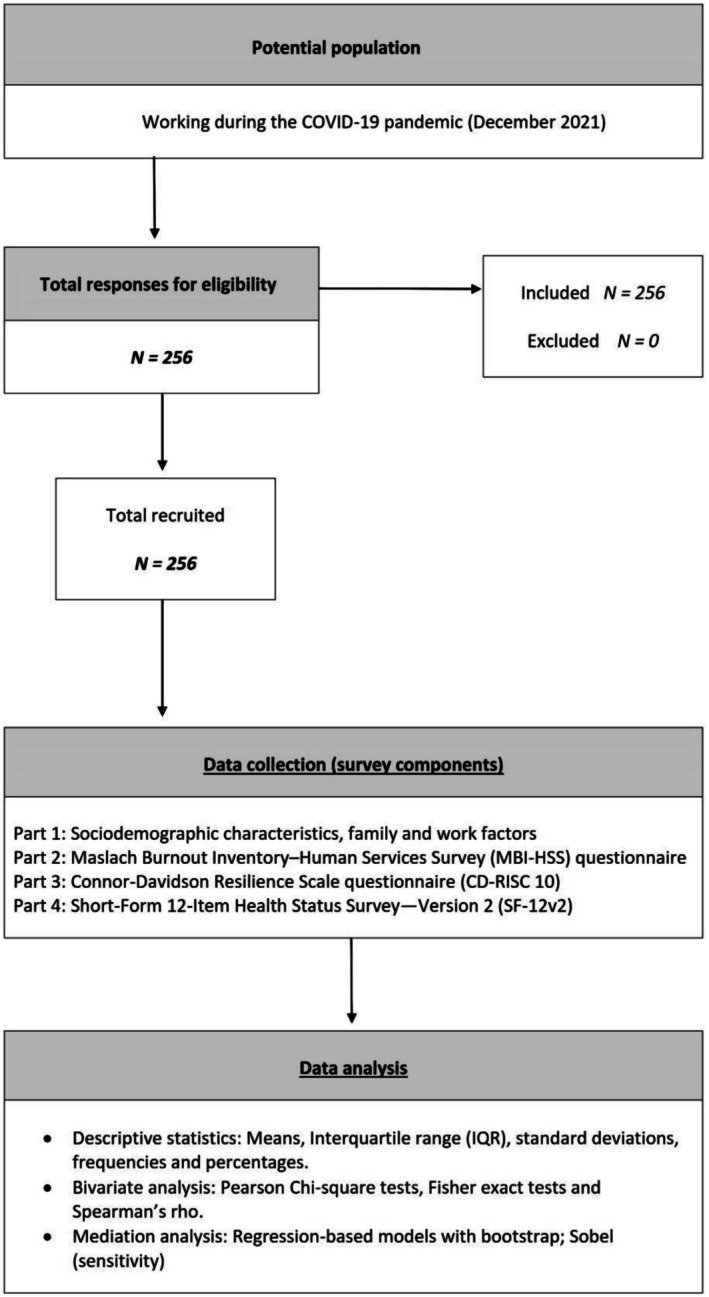
Study design.

The survey protocol was designed following the recommendations of Boynton and Greenhalgh (2004). A survey with four sections was designed to collect information. The first section gathered sociodemographic characteristics, family, and work‐related factors affecting nurses. The other three sections consisted of: the Maslach Burnout Inventory–Human Services Survey (MBI‐HSS) (Gil‐Monte 2005), the Connor‐Davidson Resilience Scale (CD‐RISC 10) (Campbell‐Sills and Stein 2007), and the Short‐Form 12‐Item Health Status Survey—Version 2 (SF‐12v2) (Monteagudo Piqueras et al. 2011) (Figure 1).

#### 3.4.1 Sociodemographic, Work, Social, and Family Characteristics

Data were collected on age, gender, educational level, workplace, employment contract, job tenure, direct contact with COVID‐19 patients, and family responsibilities. To validate the content and ensure the relevance and clarity of the items, the questionnaire underwent a face and content validation process. This process included evaluation by 10 experts—practicing nurses and educators—who reviewed each item to confirm its pertinence and comprehensibility. After incorporating their suggestions, the Content Validity Index (CVI) was calculated, obtaining values of 0.92 for clarity and 0.98 for relevance.

#### 3.4.2 Connor‐Davidson Resilience Scale (CD‐RISC 10)

The Connor‐Davidson Resilience Scale (CD‐RISC‐10) was used to measure the perceived level of resilience among nurses (Campbell‐Sills and Stein 2007). The instrument contains 10 items that assess the ability to adapt and recover in the face of adverse situations, including aspects such as flexibility, perseverance, and self‐confidence. Nurses rate their degree of agreement with the given items using a 5‐point Likert scale ranging from 0 (not at all true) to 4 (true nearly all the time). The total score is obtained by summing the responses to the 10 items, with a scoring range from 0 to 40, where higher scores indicate higher levels of resilience. The CD‐RISC‐10 has been shown to be a valid and reliable measure for assessing resilience in clinical practice (Windle et al. 2011). The Cronbach's alpha score of CD‐RISC‐10 was 0.85 in previous studies (Campbell‐Sills and Stein 2007), and similar values were obtained in adapted and validated Spanish versions, as shown in studies by Notario‐Pacheco et al. (2011) and García‐León et al. (2019).

#### 3.4.3 Maslach Burnout Inventory–Human Services Survey (MBI‐HSS)

The questionnaire used was developed and previously validated in Spanish according to the translation‐back translation procedure (Brislin 1980; Manso‐Pinto 2006; Maslach and Jackson 2012). Its application allows identifying levels of work stress and emotional exhaustion among nurses. It has 22 questions divided into 3 subcategories: Emotional Exhaustion (EE), Depersonalisation (DP), and Personal Accomplishment (PA). In the EE dimension, 9 items are associated with fatigue, tiredness, and decreased emotional energy due to work. In the DP dimension, 5 items are related to an individual's behaviour with a cold and impersonal attitude towards the people they attend to. In the PA dimension, 8 items identify situations where a person has feelings of competence and effectiveness in performing work (El Haj et al. 2020; Kakemam et al. 2021; Manso‐Pinto 2006). Each item has a 7‐point Likert scale, indicating the frequency with which they have experienced the described situation, ranging from 0 (never) to 6 (every day). Table 1 summarises the scoring criteria used to classify burnout dimensions in this study.

**TABLE 1:** Scoring criteria for the Maslach Burnout Inventory–human services survey (MBI‐HSS).

Aspect evaluated	Items to evaluate	Burnout cutoff scores
Emotional Exhaustion (EE)	1‐2‐3‐6‐8‐13‐14‐16‐20	More than 26
Depersonalisation (DP)	5‐10‐11‐15‐22	More than 9
Personal Accomplishment (PA)	4‐7‐9‐12‐17‐18‐19‐21	Less than 34

#### 3.4.4 Short‐Form 12‐Item Health Status Survey—Version 2 (SF‐12v2)

The SF‐12v2 is a brief instrument designed to assess health‐related quality of life (Vilagut et al. 2005). It evaluates two components: the physical component reflects physical health status, and the mental component evaluates psychological and emotional well‐being. The psychometric properties of the SF‐12v2 have been examined and validated in various international studies (Cheak‐Zamora et al. 2009; Jayasinghe et al. 2009; Lam et al. 2005; Montazeri et al. 2011), supporting its reliability and validity in a variety of population groups. The Spanish version has been validated in Spain (Vilagut et al. 2005; Monteagudo Piqueras et al. 2011). The dimension scores and component summary scores were calculated using the standard SF‐12v2 scoring algorithm and norm‐based scoring based on US general population means, standard deviations, and factor score coefficients (Ware Jr. et al. 2002; Ware et al. 1996). For more straightforward interpretation, norm‐based scoring generates scores for each component with a mean of 50 and a standard deviation of 10 in the reference population. This approach allows direct comparisons: a score of 50 indicates average health status, while scores above or below this point reflect better or poorer health, respectively. For instance, a physical component score of 40 indicates below‐average physical health, whereas a score of 60 reflects above‐average status (Ware et al. 1996). Because SF‐12v2 norm‐based scoring is commonly reported using US weights (mean = 50, SD = 10), we used this approach to facilitate comparison with international literature. However, applying US weights/norms to a regional Spanish sample may affect absolute score interpretation; therefore, results should be interpreted cautiously and primarily in terms of associations rather than normative classification.

### 3.5 Data Analysis

Statistical analyses were performed using SPSS version 26 (IBM Corp., Armonk, NY, USA). Mediation models were estimated in R (R Core Team 2020; version 3.6.3). Normality was assessed using the Shapiro–Wilk test. Descriptive statistics are presented as means, standard deviations (SD), medians and interquartile ranges (IQR), and frequencies as appropriate. Group differences were examined using Mann–Whitney *U* tests (two groups), chi‐square or Fisher's exact tests (categorical variables), and Spearman's rho for associations among continuous variables. Comparisons across more than two independent groups were conducted using Kruskal–Wallis tests. Mediation was examined using regression‐based models reporting total (c), direct (c'), and indirect (a × b) effects. Indirect effects were evaluated using nonparametric percentile bootstrap 95% confidence intervals (5000 resamples) as the primary inferential criterion (Preacher and Hayes 2004; MacKinnon et al. 2007). Sobel tests were computed as secondary sensitivity checks and were not used as the primary basis for inference (Sobel 1982). To address potential confounding, sensitivity models additionally adjusted for age and sex (Table S1). Age and sex were selected a priori as key sociodemographic covariates; residual confounding by occupational and COVID‐19‐related factors is possible. Statistical significance was established at *p* < 0.05 (two‐tailed). Given the cross‐sectional design, mediation results are interpreted as statistical indirect associations rather than evidence of causal mechanisms.

### 3.6 Ethical Considerations

The study was approved by the Ethics Committee of the University of Alicante (UA‐2021‐10‐22), Alicante, Spain. All participants gave their informed consent before completing the survey, ensuring their voluntary participation and data confidentiality.

### 3.7 Quality Assessment

The authors followed the STROBE (Strengthening the Reporting of Observational Studies in Epidemiology) checklist for cross‐sectional observational studies from EQUATOR (Enhancing the Quality and Transparency of Health Research) to ensure quality in this study (Von Elm et al. 2007).

## 4 Results

### 4.1 Sociodemographic Data

The sample consisted of 256 nursing professionals, of whom 83.6% (*N* = 214) were women and 16.4% (*N* = 42) were men. A Mann–Whitney *U* test was conducted to compare ages between women (*n* = 214) and men (*N* = 42). The results indicated no statistically significant difference in age between the two groups (*U* = 4058.5, *p* = 0.321). The mean age of women was 41.71 years (SD = 10.785), while that of men was 43.52 years (SD = 9.643). The Chi‐square test for the care level in which they work also showed no significant differences (*X* ^2^ = 3.400; *p* = 0.493).

Table 2 shows the sociodemographic, occupational, and family characteristics of the participants according to their workplace. Significant differences were found in the level of academic studies without specialisation among different work settings. In hospital care, the proportion of nurses without specialisation (*N* = 126) was significantly higher compared to other workplaces. Pearson's chi‐square test confirmed a significant association between the level of studies without specialisation and the workplace (χ^2^ = 33.681; df = 12; *p* = 0.0008), indicating that the absence of specialisation is more prevalent in hospital care.

**TABLE 2:** Sample description.

Workplace	Hospital care		Primary care		Social‐health center		Emergency service		Public health	
	*N*	%	*N*	%	*N*	%	*N*	%	*N*	%
Participating nurses	187	73.05	50	19.53	3	1.17	13	5.08	3	1.17
Gender										
Female	157	83.96	43	86	3	100	9	69.23	2	66.67
Male	30	16.04	7	14	0	0	4	30.77	1	33.33
Level of academic studies										
Nurse without specialisation	126	67.38	23	46	2	66.67	5	38.46	2	66.67
With specialisation	17	9.09	13	26	0	0	0	0	0	0
Master's degree	37	19.79	12	24	1	33.33	8	61.54	0	0
Doctorate	7	3.74	2	4	0	0	0	0	1	33.33
Employment contract										
Permanent position	65	34.76	18	36	1	33.33	4	30.77	1	33.33
Interim/vacant	73	39.04	17	34	0	0	3	23.08	0	0
Temporary contract	39	20.86	15	30	2	66.67	5	38.46	2	66.67
Part‐time temporary contract	10	5.35	0	0	0	0	1	7.69	0	0
Years of Experience										
< 1 year	2	1.07	0	0	0	0	0	0	0	0
1–5 years	28	14.97	10	20	1	33.33	1	7.69	0	0
5–10 years	21	11.23	8	16	1	33.33	2	15.38	0	0
10–15 years	28	14.97	5	10	0	0	3	23.08	0	0
15–20 years	40	21.39	6	12	1	33.33	2	15.38	0	0
20–25 years	29	15.51	8	16	0	0	0	0	0	0
25–30 years	15	8.02	6	12	0	0	3	23.08	2	66.67
30–35 years	14	7.49	1	2	0	0	2	15.38	1	33.33
35–40 years	5	2.67	3	6	0	0	0	0	0	0
> 40 years	5	2.67	2	4	0	0	0	0	0	0
Tenure in current position										
< 5 years	98	52.41	30	60	3	100	5	38.46	2	66.67
5–10 years	39	20.86	10	20	0	0	1	7.69	0	0
10–15 years	22	11.76	4	8	0	0	1	7.69	0	0
15–20 years	19	10.16	2	4	0	0	0	0	1	33.33
20–25 years	5	2.67	2	4	0	0	4	30.77	0	0
25–30 years	0	0	0	0	0	0	1	7.69	0	0
30–35 years	1	0.53	1	2	0	0	1	7.69	0	0
35–40 years	3	1.6	1	2	0	0	0	0	0	0
COVID‐19 infection										
Yes	45	24.06	11	22	0	0	1	7.69	1	33.33
No	119	63.64	36	72	3	100	11	84.62	2	66.67
Don't know	23	12.3	3	6	0	0	1	7.69	0	0
Family responsibilities (children and/or dependents)										
Yes	113	60.43	31	62	3	100	11	84.62	3	100

Furthermore, when examining the relationship between the level of studies without specialisation and gender in hospital care compared to other work environments, a significant association was found using Fisher's exact test (*p* = 0.047).

Statistical analysis using Pearson's chi‐square test revealed significant differences in tenure in the current position among different care levels (χ^2^ = 246.19; df = 36; *p* < 0.001). In hospital care (*N* = 187), the majority of professionals (*N* = 98; 52.4%) had less than 5 years of tenure in their current position, contrasting with other care levels where tenure is longer.

Differences in COVID‐19 infection between women and men in different care levels were evaluated. Applying Fisher's exact test to determine the association between gender and COVID‐19 infection in each level, it was found that only in primary care were there significant differences between men and women (*p* = 0.047). In this setting, the proportion of nursing professionals who reported not having been infected with COVID‐19 was significantly higher in men than in women, indicating that women had a higher infection rate in this care level.

Table 3 presents descriptive statistics for the CD‐RISC‐10 assessments, the MBI‐HSS dimensions, and the SF‐12v2 components by age group; *p* values correspond to the overall contrast between age groups.

**TABLE 3:** Descriptive statistics of the MBI‐HSS, CD‐RISC‐10, and SF‐12v2 scales by age groups.

Measure	Age groups					*p*
	23–29	30–39	40–49	50–59	> 60	
	Median [IQR]	Median [IQR]	Median [IQR]	Median [IQR]	Median [IQR]	
Connor‐Davidson Resilience Scale (CD‐RISC)						
Resilience	26 [5]	28 [7]	30 [9]	27 [9]	25 [10]	0.00923
Maslach Burnout Inventory–Human Services Survey (MBI‐HSS)						
Emotional exhaustion	26 [16]	24 [27]	23 [20]	30 [24]	32 [19]	0.553
Depersonalisation	6 [8]	8 [11]	4 [7]	4 [8]	6.5 [11]	0.0132
Personal accomplishment	34 [13]	34 [10]	38 [14]	33 [12]	33 [11]	0.514
Short‐form health survey SF‐12v2						
Physical health	54.2 [11]	50.3 [12]	52.7 [8]	46.6 [17]	43.5 [21]	3.64e‐05
Mental health	32.9 [11]	40.2 [19]	43.9 [18]	41 [22]	37 [11]	0.0323

Abbreviation: Interquartile Range (IQR).

### 4.2 Description of Questionnaire Variables

Table 4 shows the median score and interquartile range (IQR) for all measures. Cronbach's alpha is reported for the CD‐RISC‐10 and MBI‐HSS subscales only, with values ranging from 0.75 to 0.91 in this sample, indicating good internal consistency. For the SF‐12v2 component summary scores, psychometric quality was interpreted on the basis of the instrument's established validation literature rather than internal consistency coefficients.

**TABLE 4:** Descriptive statistics of the MBI‐HSS, CD‐RISC‐10, and SF‐12v2 scales by gender.

Measure	Items	Women Median [IQR]	Men Median [IQR]	Total Median [IQR]	Cronbach's α
Connor‐Davidson Resilience Scale (CD‐RISC‐10)					
Resilience	10	27 [7]	30 [8]	28 [8]	0.91
Maslach Burnout Inventory–Human Services Survey (MBI‐HSS)					
Emotional exhaustion	9	26 [20]	22 [31]	26 [22]	0.91
Depersonalisation	5	5 [8]	8 [11]	5 [9]	0.75
Personal accomplishment	8	35 [11]	35 [17]	35 [12]	0.81
Short‐form health survey SF‐12v2					
Physical health	6	51 [13]	54 [7]	52 [12]	
Mental health	6	39 [20]	42 [18]	40 [20]	

*Note:* Cronbach's α is reported for the CD‐RISC‐10 and MBI‐HSS subscales only; SF‐12v2 component summary scores were interpreted on the basis of published psychometric evidence rather than internal consistency coefficients.

Abbreviation: Interquartile Range (IQR).

### 4.3 Resilience Levels

**FIGURE 2:**
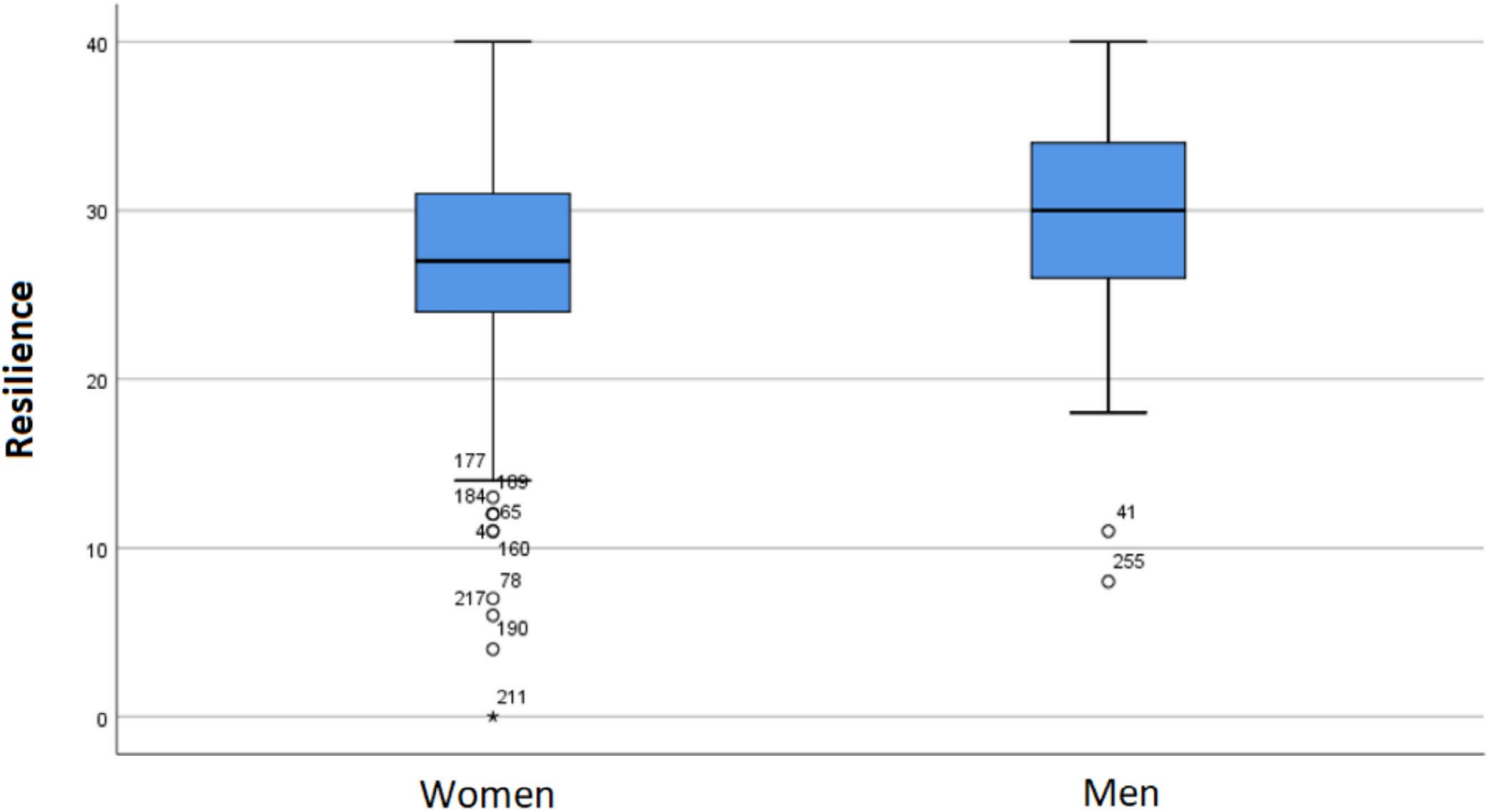
Level of resilience (CD‐RISC) by gender.

Regarding resilience, measured with the Spanish version of the CD‐RISC 10 (Campbell‐Sills and Stein 2007; Notario‐Pacheco et al. 2011), the data indicate that medians differ between women and men. As shown in Figure 2, women presented a median of 27 [IQR = 7], while men showed a median of 30 [IQR = 8]. The difference was statistically significant (*U* = 3303; *p* = 0.006), indicating that men had significantly higher levels of resilience. These results are comparable to those found in previous studies conducted in Spain during the COVID‐19 pandemic. For example, Jiménez‐Fernández et al. (2022), in their study with frontline nurses in Madrid, reported a mean total resilience of 28.75 (SD = 5.50), indicating medium to low levels of resilience. This value is similar to the total median obtained in our sample (28), suggesting that nurses during the pandemic present moderate levels of resilience.

### 4.4 Correlations Between Questionnaire Variables (Resilience, Burnout, Mental and Physical Health) Using Spearman's Rho

Correlations between resilience, the dimensions of burnout (emotional exhaustion, depersonalisation, and personal accomplishment), and physical and mental health were analysed using Spearman's rho. The results are presented in Table 5.

**TABLE 5:** Correlations between variables using Spearman's rho.

Variables	Resilience	Physical health	Mental health
Connor‐Davidson Resilience Scale (CD‐RISC‐10)			
Resilience		0.138*	0.418**
Maslach Burnout Inventory–Human Services Survey (MBI‐HSS)			
Emotional exhaustion	−0.373**	−0.307**	−0.675**
Depersonalisation	−0.169**	−0.125*	−0.420**
Personal accomplishment	0.392**	0.072	0.330**
Short‐form health survey SF‐12v2			
Physical health	0.138*		0.021
Mental health	0.418**	0.021	

*Note:* Spearman's rho coefficients.

* *p* ≤ 0.05.

** *p* ≤ 0.01 (two‐tailed).

Spearman correlation coefficients ranged between −0.675 and 0.508, indicating small to strong monotonic associations among the variables. In particular, resilience was negatively associated with emotional exhaustion (*ρ* = −0.373; *p* < 0.001) and depersonalisation (*ρ* = −0.169; *p* = 0.007), and positively associated with personal accomplishment (*ρ* = 0.392; *p* < 0.001).

Additionally, resilience was positively associated with mental health (*ρ* = 0.418; *p* < 0.001) and weakly associated with physical health (*ρ* = 0.138; *p* = 0.027). These findings suggest that higher levels of resilience were associated with lower levels of burnout in its negative dimensions, greater feelings of personal accomplishment, and better perceived mental health.

Emotional exhaustion showed a strong negative association with mental health (*ρ* = −0.675; *p* < 0.001) and a moderate negative association with physical health (*ρ* = −0.307; *p* < 0.001). This indicates that higher levels of emotional exhaustion were associated with poorer mental and physical health. Depersonalisation was also negatively associated with mental health (*ρ* = −0.420; *p* < 0.001) and weakly associated with physical health (*ρ* = −0.125; *p* = 0.046). Personal accomplishment was positively associated with mental health (*ρ* = 0.330; *p* < 0.001) but did not show a statistically significant bivariate association with physical health (*ρ* = 0.072; *p* = 0.248).

Additionally, emotional exhaustion and depersonalisation were positively associated (*ρ* = 0.508; *p* < 0.001), and both were negatively associated with personal accomplishment (emotional exhaustion: *ρ* = −0.344; *p* < 0.001; depersonalisation: *ρ* = −0.322; *p* < 0.001). These results indicate that the negative dimensions of burnout were interrelated and inversely associated with the sense of personal accomplishment.

Overall, the negative dimensions of burnout were associated with poorer mental and physical health, while resilience and personal accomplishment were associated with better mental health. Physical health showed weaker bivariate associations than mental health in this sample.

### 4.5 Mediation Analysis of Resilience

Due to the cross‐sectional design of the study, the identified indirect effects are exploratory and associative and do not imply causality. Mediation models were estimated using regression‐based mediation analysis to test whether resilience statistically accounted for associations between burnout dimensions and health outcomes. Indirect effects (a × b) were evaluated using nonparametric percentile bootstrap 95% confidence intervals (5000 resamples) as the primary inferential approach; Sobel tests were computed as secondary sensitivity checks.

Table 6 reports unstandardised regression coefficients (B) for paths a, b, c' and total c, and the bootstrap 95% confidence interval for a × b. Sensitivity analyses adjusting for age and sex yielded a similar pattern for the indirect effects (Table S1).

**TABLE 6:** Mediation analysis of resilience in the relationship between burnout and health.

Independent variable (X)	Outcome (Y)	Mediator (M)	Path a: (B, *p*)	Path b: (B, *p*)	Direct effect c' (B, *p*)	Indirect effect a × b (B)	95% CI (bootstra*p*)	Total effect c (B, *p*)
Emotional exhaustion	Physical Health	Resilience	−0.1738 (< 0.001)	0.1231 (0.135)	−0.1968 (< 0.001)	−0.0214	[−0.0561; 0.0088]	−0.2182 (< 0.001)
Emotional exhaustion	Mental Health	Resilience	−0.1738 (< 0.001)	0.2710 (< 0.001)	−0.5329 (< 0.001)	−0.0471	[−0.0925; −0.0145]	−0.5800 (< 0.001)
Depersonalisation	Physical Health	Resilience	−0.1858 (0.010)	0.2288 (0.005)	−0.1777 (0.060)	−0.0425	[−0.1006; −0.0038]	−0.2202 (0.020)
Depersonalisation	Mental Health	Resilience	−0.1858 (0.010)	0.5249 (< 0.001)	−0.7161 (< 0.001)	−0.0975	[−0.2020; −0.0180]	−0.8137 (< 0.001)
Personal accomplishment	Mental Health	Resilience	0.2963 (< 0.001)	0.5038 (< 0.001)	0.2478 (0.003)	0.1493	[0.0725; 0.2369]	0.3971 (< 0.001)
Personal accomplishment	Physical Health	Resilience	0.2963 (< 0.001)	0.2565 (0.003)	−0.0067 (0.922)	0.0760	[0.0211; 0.1464]	0.0693 (0.279)

*Note:* Coefficients are unstandardised regression coefficients (B). Indirect effects (a × b) were evaluated using percentile bootstrap 95% confidence intervals (5000 resamples). The indirect effect was considered statistically significant when the 95% CI did not include 0. Resilience = Connor‐Davidson Resilience Scale (CD‐RISC‐10); Maslach Burnout Inventory–Human Services Survey (MBI‐HSS); Physical Health and Mental Health = components of the Short‐form Health Survey SF‐12v2.

Indirect effects involving physical health were more heterogeneous and generally smaller than those for mental health and should be interpreted cautiously.

#### 4.5.1 Resilience as a Mediating Variable in the Relationship With Emotional Exhaustion and Physical and Mental Health

##### 4.5.1.1 Resilience, Emotional Exhaustion, and Physical Health

**FIGURE 3:**
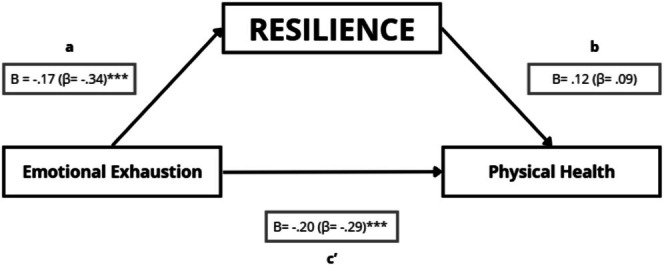
Mediation model testing whether resilience statistically mediates the association between emotional exhaustion and physical health. Path a represents the association between emotional exhaustion and resilience; path b represents the association between resilience and physical health (adjusted for emotional exhaustion); and path c' represents the direct effect of emotional exhaustion on physical health (adjusted for resilience). Unstandardised regression coefficients (B) are shown, with standardised coefficients (β) in parentheses. *p ≤ 0.05; **p ≤ 0.01; ***p ≤ 0.001.

The analysis revealed a significant negative association between emotional exhaustion and resilience (path a: *B* = −0.17; *p* < 0.001), indicating that higher emotional exhaustion was associated with lower resilience (Figure 3). Emotional exhaustion was directly associated with poorer physical health (direct effect c': *B* = −0.20; *p* < 0.001). Resilience was positively but not significantly associated with physical health (path b: *B* = 0.12; *p* = 0.135). The bootstrapped indirect effect through resilience was small and not statistically significant (a × b = −0.021; 95% bootstrap CI [−0.056, 0.009]); the Sobel test was also not significant *Z* = −1.45 (*p* = 0.146). Overall, these findings are not consistent with a statistically significant indirect association through resilience for physical health in this model (Figure 3; Table 6).

##### 4.5.1.2 Resilience, Emotional Exhaustion, and Mental Health

**FIGURE 4:**
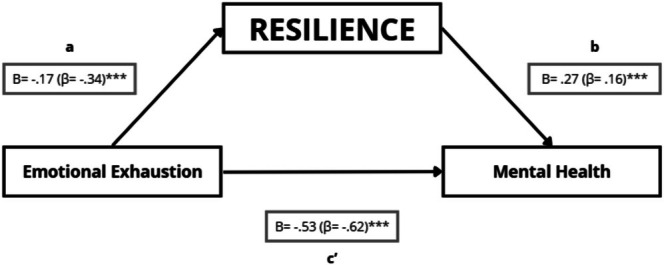
Mediation model testing whether resilience statistically mediates the association between emotional exhaustion and mental health. Path a represents the association between emotional exhaustion and resilience; path b represents the association between resilience and mental health (adjusted for emotional exhaustion); and path c' represents the direct effect of emotional exhaustion on mental health (adjusted for resilience). Unstandardised regression coefficients (B) are shown, with standardised coefficients (β) in parentheses. *p ≤ 0.05; **p ≤ 0.01; ***p ≤ 0.001.

Emotional exhaustion was negatively associated with resilience (path a: *B* = −0.17; *p* < 0.001), indicating that higher emotional exhaustion was associated with lower resilience (Figure 4). Resilience was positively associated with mental health after accounting for emotional exhaustion (path b: *B* = 0.27; *p* < 0.001). Emotional exhaustion remained negatively associated with mental health when resilience was included in the model (direct effect c': *B* = −0.53; *p* < 0.001).

The indirect effect through resilience was negative and statistically significant (a × b = −0.047; 95% bootstrap CI [−0.093; −0.015]), indicating that resilience statistically accounted for part of the association between emotional exhaustion and mental health (Table 6). As a secondary sensitivity check, the Sobel test was also significant (*Z* = −2.90; *p* = 0.004).

#### 4.5.2 Resilience as a Mediating Variable in the Relationship With Depersonalisation and Physical and Mental Health

##### 4.5.2.1 Resilience, Depersonalisation, and Physical Health

**FIGURE 5:**
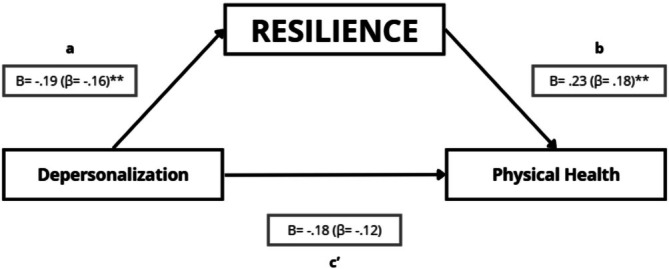
Mediation model testing whether resilience statistically mediates the association between depersonalisation and physical health. Path a represents the association between depersonalisation and resilience; path b represents the association between resilience and physical health (adjusted for depersonalisation); and path c' represents the direct effect of depersonalisation on physical health (adjusted for resilience). Unstandardised regression coefficients (B) are shown, with standardised coefficients (β) in parentheses. *p ≤ 0.05; **p ≤ 0.01; ***p ≤ 0.001.

The mediation model indicated that depersonalisation was negatively associated with resilience (path a: *B* = −0.19; *p* = 0.010), and resilience was positively associated with physical health after controlling for depersonalisation (path b: *B* = 0.23; *p* = 0.005) (Figure 5). The direct effect of depersonalisation on physical health controlling for resilience was not statistically significant (path c': *B* = −0.18; *p* = 0.060). However, the bootstrap indirect effect (a × b) was negative and statistically significant (95% CI did not include zero), suggesting a small indirect association between depersonalisation and physical health through resilience (Table 6). As a secondary sensitivity check, the Sobel test was borderline (*Z* = −1.91; *p* = 0.06). In sensitivity models adjusting for age and sex, the indirect effect remained statistically significant and the direct association between depersonalisation and physical health was stronger (Table S1).

##### 4.5.2.2 Resilience, Depersonalisation, and Mental Health

**FIGURE 6:**
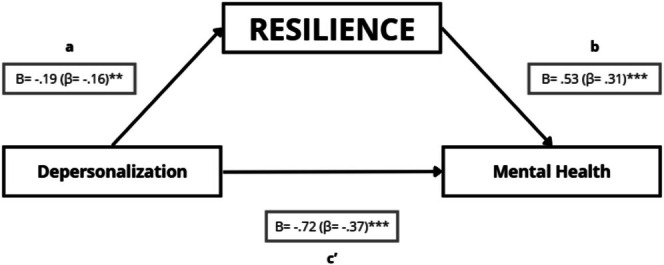
Mediation model testing whether resilience statistically mediates the association between depersonalisation and mental health. Path a represents the association between depersonalisation and resilience; path b represents the association between resilience and mental health (adjusted for depersonalisation); and path c' represents the direct effect of depersonalisation on mental health (adjusted for resilience). Unstandardised regression coefficients (B) are shown, with standardised coefficients (β) in parentheses. *p ≤ 0.05; **p ≤ 0.01; ***p ≤ 0.001.

In the mediation analysis of depersonalisation and mental health, depersonalisation was negatively associated with resilience (path a: *B* = −0.19; *p* = 0.010). Resilience was positively associated with mental health after accounting for depersonalisation (path b: *B* = 0.53; *p* < 0.001). Depersonalisation also showed a negative direct effect on mental health controlling for resilience (path c': *B* = −0.72; *p* < 0.001). The indirect effect through resilience was negative (a × b = −0.0975), and its bootstrap 95% confidence interval excluded zero (95% CI: [−0.2020; −0.0180]), indicating a statistically significant indirect association (Table 6; Figure 6). As a secondary sensitivity check, the Sobel test was also significant (*Z* = −2.36; *p* = 0.018).

#### 4.5.3 Resilience as a Mediating Variable in the Relationship With Personal Accomplishment and Physical and Mental Health

##### 4.5.3.1 Resilience, Personal Accomplishment, and Mental Health

**FIGURE 7:**
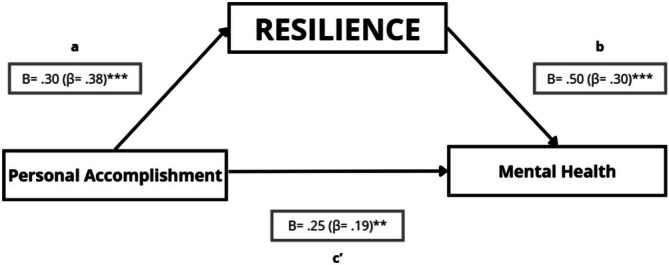
Mediation model testing whether resilience statistically accounts for the association between personal accomplishment and mental health. Path a represents the association between personal accomplishment and resilience; path b represents the association between resilience and mental health (adjusted for personal accomplishment); and path c' represents the direct effect of personal accomplishment on mental health (adjusted for resilience). Unstandardised regression coefficients (B) are shown, with standardised coefficients (β) in parentheses. *p ≤ 0.05; **p ≤ 0.01; ***p ≤ 0.001.

In the mediation model examining personal accomplishment, resilience, and mental health, personal accomplishment was positively associated with resilience (path a: *B* = 0.30; *p* < 0.001). Resilience was positively associated with mental health when adjusting for personal accomplishment (path b: *B* = 0.50; *p* < 0.001). Personal accomplishment also showed a positive direct association with mental health after accounting for resilience (path c': *B* = 0.25; *p* = 0.003). The indirect effect through resilience was positive and statistically significant (a × b = 0.15; 95% bootstrap CI [0.07, 0.24]), consistent with a partial indirect association via resilience (Figure 7; Table 6). As a secondary sensitivity check, the Sobel test was also statistically significant (*Z* = 3.93; *p* < 0.001).

##### 4.5.3.2 Resilience, Personal Accomplishment, and Physical Health

**FIGURE 8:**
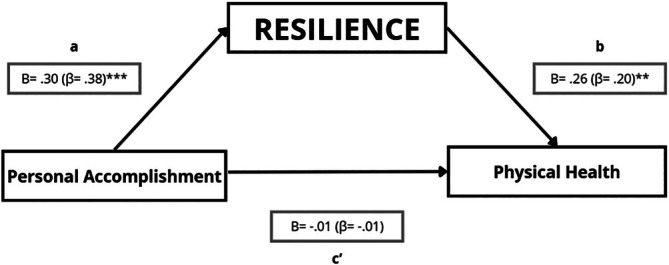
Mediation model testing whether resilience statistically accounts for the association between personal accomplishment and physical health. Path a represents the association between personal accomplishment and resilience; path b represents the association between resilience and physical health (adjusted for personal accomplishment); and path c' represents the direct effect of personal accomplishment on physical health (adjusted for resilience). Unstandardised regression coefficients (B) are shown, with standardised coefficients (β) in parentheses. *p ≤ 0.05; **p ≤ 0.01; ***p ≤ 0.001.

A mediation model was additionally estimated for personal accomplishment, resilience, and physical health. Personal accomplishment was positively associated with resilience (path a: *B* = 0.30; *p* < 0.001), and resilience was positively associated with physical health after accounting for personal accomplishment (path b: *B* = 0.26; *p* = 0.003). Although the total association between personal accomplishment and physical health was small and not statistically significant (c: *B* = 0.07; *p* = 0.279), the indirect effect through resilience was positive and statistically significant (a × b = 0.0760; 95% bootstrap CI [0.0211; 0.1464]). The direct effect controlling for resilience was close to zero and not statistically significant (c': *B* = −0.0067; *p* = 0.922). As a secondary sensitivity check, the Sobel test was also significant (*Z* = 2.69; *p* = 0.007) (Figure 8). In sensitivity models adjusting for age and sex, the indirect effect remained statistically significant (Table S1).

## 5 Discussion

This study analysed indirect associations through resilience in the relationship between burnout dimensions and physical and mental health in a sample of nurses from the Valencian Community in December 2021 during the COVID‐19 pandemic. Results are consistent with resilience being associated with better mental health and statistically accounting for part of the association between burnout dimensions and mental health. Men showed higher resilience than women, and resilience differed across age groups.

Most participants were women, which is consistent with the gender distribution in the nursing profession both nationally and internationally (National Institute of Statistics 2022). The average age of the participants indicates a workforce with experience, which is essential for providing quality care (McHugh and Ma 2014). The average tenure of 16 years suggests that nurses have developed fundamental skills and knowledge for clinical practice (Arsat et al. 2023).

Significant differences were found in resilience levels between men and women, with men showing higher resilience. This aligns with some studies suggesting that women may face greater contextual stressors, such as additional domestic responsibilities, affecting their resilience capacity (Lowe et al. 2021; Sardella et al. 2022). However, other studies have not found significant differences based on gender (Rivas et al. 2021), indicating the need for further research to better understand these findings and develop specific interventions.

Regarding the dimensions of burnout, nurses presented elevated levels of emotional exhaustion, which may be indicative of initial phases of burnout syndrome, according to established cutoff points (Gil‐Monte 2005). Depersonalisation was also present, although at moderate levels, and personal accomplishment remained above the risk threshold, suggesting that despite exhaustion, nurses maintain a sense of professional efficacy. These results are consistent with previous studies that have identified high levels of burnout among nursing staff during the pandemic (Galanis et al. 2021; Pappa et al. 2020).

Mediation analyses supported statistically significant indirect associations through resilience primarily for mental health outcomes. Higher emotional exhaustion and depersonalisation were associated with lower resilience, and lower resilience was associated with poorer mental health, yielding negative indirect effects (a × b < 0). In contrast, personal accomplishment showed a positive indirect effect on mental health through resilience (a × b > 0) (Table 6). These findings should be interpreted as exploratory indirect associations given the cross‐sectional design. Prior nursing research has described resilience as a personal resource associated with better psychological health and a potential buffering factor in contexts of burnout and chronic work stress (García‐Izquierdo et al. 2018; Hart et al. 2014). Similar cross‐sectional studies have reported indirect effects of resilience in relation to occupational stress and mental health in nurses and healthcare workers (Arrogante 2014; Chen et al. 2022; Galdames et al. 2024; Serrão et al. 2021).

For physical health, evidence for indirect associations through resilience was weaker and more mixed: no indirect effect was observed for emotional exhaustion, whereas depersonalisation showed a small negative indirect effect through resilience, and personal accomplishment showed a positive indirect effect through resilience (Table 6), which persisted in sensitivity models adjusting for age and sex (Table S1). Other factors (e.g., working conditions, social support, and occupational exposures) may also contribute to physical health outcomes (Burdorf et al. 2020; Dewa et al. 2014).

In relation to age groups, nurses aged 40–49 years showed higher resilience and better mental and physical health. This may relate to greater experience and the development of more effective coping strategies over time (Mealer et al. 2012). Physical health tended to be lower in the older age groups, highlighting the potential value of age‐tailored occupational health strategies (Deltour et al. 2023).

The primary conceptual and practical contribution of this study lies in its focus on the late‐pandemic period (December 2021) of the COVID‐19 pandemic, a period marked by chronic exhaustion and a gradual decline in institutional support. During the first wave (March–May 2020), healthcare workers experienced high levels of burnout, post‐traumatic stress, and anxiety, exacerbated by uncertainty and shortages of material and human resources (Luceño‐Moreno et al. 2020; Morgantini et al. 2020). At that time, resilience often emerged as an immediate collective response, sustained by a strong sense of mission and team cohesion.

In the second wave (late 2020 to spring 2021), various studies described the onset of pandemic fatigue, with persistent stress linked to perceptions of limited institutional support and prolonged emotional strain (Jiménez‐García et al. 2022). Recent studies indicate that, despite lower infection rates, burnout has remained widespread, showing a sustained impact without clear signs of recovery (Serrão et al. 2021).

Evidence from previous health emergencies, such as the 2014–2016 West African Ebola outbreak, points to comparable challenges in different contexts. These crises were also associated with high levels of anxiety and stress due to occupational risks, social stigma, and limited psychosocial support (Raven et al. 2018). These examples underscore the importance of implementing early measures to enhance resilience and provide sufficient emotional and psychological support for healthcare workers. Studying resilience in late 2021 during the COVID‐19 pandemic can clarify how this capacity is maintained during prolonged crises and add to existing knowledge on burnout and coping.

Due to the cross‐sectional design, the mediation results reflect statistical associations only and do not establish causality. Although the indirect effects of resilience were statistically significant, their sizes were moderate or small. Both statistical and practical aspects should be considered when interpreting these findings. Further research is needed to better understand the clinical and occupational implications of these effects and to relate statistical estimates to their actual impact on daily nursing work.

### 5.1 Limitations and Strengths

This study has several limitations that should be considered when interpreting the results. First, its cross‐sectional design does not allow for causal conclusions or for determining the direction of the observed associations. Longitudinal designs help clarify how resilience, burnout, and health outcomes interact and evolve among nurses. Second, the convenience sample was limited to nurses in a single geographical area, which may reduce the applicability of the findings to other contexts. Because recruitment used a convenience online survey disseminated via multiple channels, the sampling denominator and response rate are unknown. To minimise duplicate submissions, response patterns were screened for potential duplicate entries and any repeated records considered compatible with duplicate participation were removed before analysis; however, as with any open online survey, undetected duplication cannot be ruled out completely (Eysenbach 2004). Third, the use of self‐reported online questionnaires may have introduced reporting bias. Fourth, subgroup comparisons were exploratory and may be sensitive to multiple testing. Other relevant factors, such as social support, coping strategies, or specific working conditions, were not included, although they may also affect burnout and resilience. Personality traits were not considered either, despite existing evidence of their possible moderating or mediating role in psychological well‐being (Pakou et al. 2024). Including these aspects in future studies would provide a more complete view of the factors influencing nurses' mental health. Fifth, mediation results should be interpreted as statistical indirect associations; cross‐sectional mediation analyses can differ from longitudinal processes (Maxwell and Cole 2007). Finally, SF‐12v2 component scores were computed using US norm‐based scoring; therefore, absolute component values should be interpreted cautiously in this Spanish regional sample.

Information on the severity of COVID‐19 infection among participants was also not collected. Severe cases can have long‐term consequences for both physical and mental health. They may act as confounding factors, making it difficult to assess the extent to which infection severity influenced the results for emotional exhaustion, resilience, and health status. Future work should gather detailed clinical information to examine this variable more precisely.

Despite these limitations, this study makes valuable contributions. It is one of the few that analyses the mediating role of resilience between burnout and both physical and mental health in nurses during the later stage of the COVID‐19 pandemic, adding updated data on a factor with implications for care quality and workforce well‐being.

The use of validated and widely recognised instruments adds methodological consistency and supports the reliability of the measures used. Finally, including nurses from different levels of care and service settings provides a broader perspective on the issue, thereby strengthening the relevance of the findings for various areas of nursing practice.

### 5.2 Recommendations for Further Research

Future research should include additional variables such as personality traits, as they may significantly influence the relationship between burnout and health, providing a more comprehensive understanding of the factors affecting nurses' well‐being. Additionally, it is recommended to conduct longitudinal studies to establish causal relationships and observe how resilience and burnout evolve over time. Resilience‐support strategies are plausible, but only intervention studies and prospective cohort designs can clarify whether related measures produce measurable effects in clinical practice.

Expanding the sample to different regions and work contexts would improve the generalisation of the results. Finally, employing mixed methodologies that combine quantitative and qualitative approaches would enrich the understanding of these phenomena and contribute to the design of interventions.

### 5.3 Implications for Policy and Practice

The results of this study have important practical implications. Resilience‐support programs and organisational measures aimed at reducing burnout are plausible strategies, but their effectiveness should be evaluated using longitudinal and experimental designs. Promoting resilience training programs and developing work environments that reduce burnout may improve nurses' well‐being and, consequently, the quality of patient care (Cleary et al. 2018; Cooper et al. 2020). Moreover, given the influence of gender on resilience and burnout levels, it is essential to consider strategies that address the specific needs of men and women in the profession (Szabo et al. 2020).

Other research also highlights the role of management in improving nurses' working conditions by incorporating various measures, such as support, analysis of workload, adequate human resources, problem‐oriented meetings to resolve ongoing issues, and psychological support as needed (Sierakowska and Doroszkiewicz 2022).

## 6 Conclusions

This study found that resilience was associated with nurses' mental health in the Valencian Community in December 2021 during the COVID‐19 pandemic. Higher resilience co‐occurred with better mental health even in the presence of elevated burnout dimensions. Given the cross‐sectional design, these associations do not establish causality and should be confirmed using longitudinal or experimental studies. Additionally, factors such as gender and age influence resilience levels, indicating the need to develop personalised interventions to strengthen this capacity.

On the other hand, nurses experienced elevated levels of burnout, especially in emotional exhaustion and depersonalisation. These burnout dimensions were associated with poorer mental health. These findings reinforce the importance of addressing workplace stressors and supporting work–life balance, while recognising that the impact of specific strategies should be tested in future studies.

Although the mediation analyses showed statistically significant indirect effects of resilience, their sizes were moderate or small. Indirect associations with physical health were more limited and pathway‐specific. These findings highlight the importance of testing resilience‐focused interventions under controlled conditions. Before broader application, clinical trials or longitudinal studies should confirm whether these measures help lower burnout levels and strengthen patient care. Further studies with longitudinal designs would also help clarify the long‐term effects of pandemics on nurses and assess the effectiveness of these interventions in practice.

## 7 Relevance for Clinical Practice

Identifying resilience as a factor associated with mental health highlights the potential value of supportive strategies aimed at strengthening coping resources in the work environment. However, the effectiveness of such strategies should be evaluated using robust study designs before strong practice recommendations are made.

Healthcare institutions may consider training programs and workshops focused on resilience and stress coping. These interventions could include stress management techniques, mindfulness, promotion of healthy habits, and strategies to foster work‐life balance. Their effectiveness for improving well‐being and care‐related outcomes should be evaluated using robust study designs.

Additionally, healthcare organisations may consider recognising and addressing factors that contribute to burnout, such as excessive workloads, lack of organisational support, and adverse working conditions. Policies that promote healthy work environments, offer psychological and emotional support, and ensure adequate resources may help reduce burnout levels among nursing staff, but their impact should be evaluated empirically.

Personalising interventions, taking into account sociodemographic factors such as gender and age, may increase the relevance and acceptability of implemented strategies. For example, mentorship programs and peer support may be beneficial for younger or less experienced nurses, while professional development opportunities may enhance personal accomplishment and job satisfaction in more experienced nurses.

## Author Contributions

S.J.‐G. made substantial contributions to conception and design, or acquisition of data, or analysis and interpretation of data. S.J.‐G., E.L., F.V.‐M. involved in drafting the manuscript or revising it critically for important intellectual content. S.J.‐G., E.L., F.V.‐M. given final approval of the version to be published. Each author should have participated sufficiently in the work to take public responsibility for appropriate portions of the content. S.J.‐G., E.L., F.V.‐M. agreed to be accountable for all aspects of the work in ensuring that questions related to the accuracy or integrity of any part of the work are appropriately investigated and resolved.

## Funding

The authors have nothing to report.

## Ethics Statement

This study was conducted in accordance with the Declaration of Helsinki, and the protocol was approved by the Ethics Committee of the University of Alicante (UA‐2021‐10‐22), Alicante, Spain. We confirm that all data used in the manuscript have been legally obtained and in compliance with current ethical regulations. This study did not involve access to or use of genetic resources or biological materials regulated by the Nagoya Protocol. The fieldwork was conducted following established ethical norms, with the approval of the Ethics Committee of the University of Alicante (UA‐2021‐10‐22) and with the informed consent of all participants.

## Consent

Participants received information about the study with the invitation to participate and the access link. Participants signed an informed consent form in the questionnaire to participate in the study.

## Conflicts of Interest

The authors declare no conflicts of interest.

## Supporting information


**Table S1:** Sensitivity analysis: mediation models adjusted for age and sex Indirect effects are shown with 95% bootstrap confidence intervals (5000 resamples).

## Data Availability

The data presented in this study are available upon request from the corresponding author.
