# Supplementary material for: Resilience as a Mediator Between Burnout and Health Among Nurses During the COVID‐19 Pandemic: A Cross‐Sectional Survey in Late 2021
Source: Nurs Open. 2026 Apr 14;13(4):e70526. doi: 10.1002/nop2.70526 (PMC13079419; doi:10.1002/nop2.70526)
Supplement: Supplementary file 1 — Table S1: Sensitivity analysis: mediation models adjusted for age and sex Indirect effects are shown with 95% bootstrap confidence intervals (5000 resamples). [file NOP2-13-e70526-s001.docx]

## Supplementary Table S1. Sensitivity analysis: mediation models adjusted for age and sex

Indirect effects are shown with 95% bootstrap confidence intervals (5,000 resamples).

| **Independent variable (X)** | **Outcome (Y)** | **Indirect effect a×b (B)** | **95% CI (bootstrap)** | **Direct effect c′ (B, p)** |
| --- | --- | --- | --- | --- |
| **Emotional exhaustion** | Physical health | -0.0161 | [-0.0481; 0.0127] | -0.1963 (<.001) |
| **Emotional exhaustion** | Mental health | -0.0481 | [-0.0905; -0.0182] | -0.5330 (<.001) |
| **Depersonalization** | Physical health | -0.0396 | [-0.0941; -0.0027] | -0.2454 (.0077) |
| **Depersonalization** | Mental health | -0.1120 | [-0.2191; -0.0300] | -0.7000 (<.001) |
| **Personal accomplishment** | Mental health | 0.1521 | [0.0729; 0.2382] | 0.2450 (.0026) |
| **Personal accomplishment** | Physical health | 0.0660 | [0.0143; 0.1298] | 0.0030 (.964) |
